# Total Laparoscopic Resection of Hilar Cholangiocarcinoma Type 3b: Applying a Parachute Technique for Hepaticojejunostomy

**DOI:** 10.1245/s10434-020-09175-1

**Published:** 2020-09-30

**Authors:** Robert Sucher, Uwe Scheuermann, Daniel Seehofer

**Affiliations:** Department of Visceral, Transplant, Thoracic, and Vascular Surgery, University Clinic Leipzig, Leipzig, Germany

## Abstract

**Background:**

Laparoscopic liver resection for perihilar cholangiocarcinoma (pCCA) is still in its infancy. The biliary-enteric reconstruction represents one of the most delicate parts of this minimally invasive procedure.

**Methods:**

In this study, a 78-year old woman with perihilar cholangiocarcinoma (pCCA) type 3b underwent a hepaticojejunostomy performed by a parachute technique.

**Results:**

The operation, performed totally by minimally invasive resections, was completed in 386 min, with a blood loss of less than 400 ml and no transfusion requirements. Two intraluminal stents were placed during the hepaticojenunostomy for splinting of the biliary-enteric anastomosis. The patient required prolonged antibiotic treatment for postoperative cholangitis and finally was discharged on postoperative day 15. The histopathologic grading displayed a G 2–3 adenocarcinoma, pT3 pN0, M0, L1, V1, pN1, UICC IIIc R0, and the patient was referred to adjuvant chemotherapy.

**Conclusion:**

Resections of pCCAs, performed totally by minimally invasive techniques, may be feasible and safe for a selected group of patients. With this approach, a running-suture hepaticojejunostomy using the parachute technique represents a worthwhile strategy for biliary-enteric reconstruction.

**Electronic supplementary material:**

The online version of this article (10.1245/s10434-020-09175-1) contains supplementary material, which is available to authorized users.

Perihilar cholangiocarcinomas (pCCA) Bismuth Corlette types 3a and 3b require radical lymphadenectomy, resection of the respective hemiliver, extrahepatic bile duct resection, and biliary reconstruction by hepaticojejunostomy to achieve long-term patient survival.^1^ Although technically highly demanding, all the aforementioned critical steps of cholangiocarcinoma (CCA) surgery recently have been performed totally by minimally invasive techniques for selected groups of patients.^2^^–^4 Furthermore, a recent analysis of patients with pCCA treated in one of the leading European centers for laparoscopic liver surgery showed no oncologic inferiority of a laparoscopic resection, which might have been a major concern of surgeons preparing for the final step toward minimally invasiveness in pCCA surgery.^5^

However, from the expert’s point of view, minimally invasive pCCA surgery remains in an exploratory phase, with the conversion rate of 18.8% reaching the rate generally reported for major laparoscopic hepatectomies.^6^ Nevertheless, the approach did not have an adverse impact on outcome, as recently reported in another study.^7^ Furthermore, some operational steps, such as the biliary-enteric anastomosis, still may be performed in an old-school manner through a larger service incision. Formal lymphadenectomy is mandatory in the process of pCCA surgery because lymph node status is one of the most important factors for long-term outcome.^8^ In addition, a recent study by Ratti et al.^9^ demonstrated that laparoscopic lymphadenectomy is a valid option for patients with biliary cancer, and that a minimally invasive surgical approach does not compromise the accuracy or outcome of lymph node dissection.

We recently implemented a minimally invasive hepatobiliary surgical program into our daily routine, and propelled by encouraging results from specialized centers, we currently aim to expand our service to selected patients with pCCA. According to our current perspective, for laparoscopic pCCA surgery, the vascular inflow of the remaining liver segments must not be infiltrated by tumor. This also includes the bifurcation of the portal vein, which commonly is adherent to or encased by central tumors, and for oncologic reasons might require open resection and reconstruction.

## Materials and Methods

### Perioperative Patient Examination

**Fig. 1:**
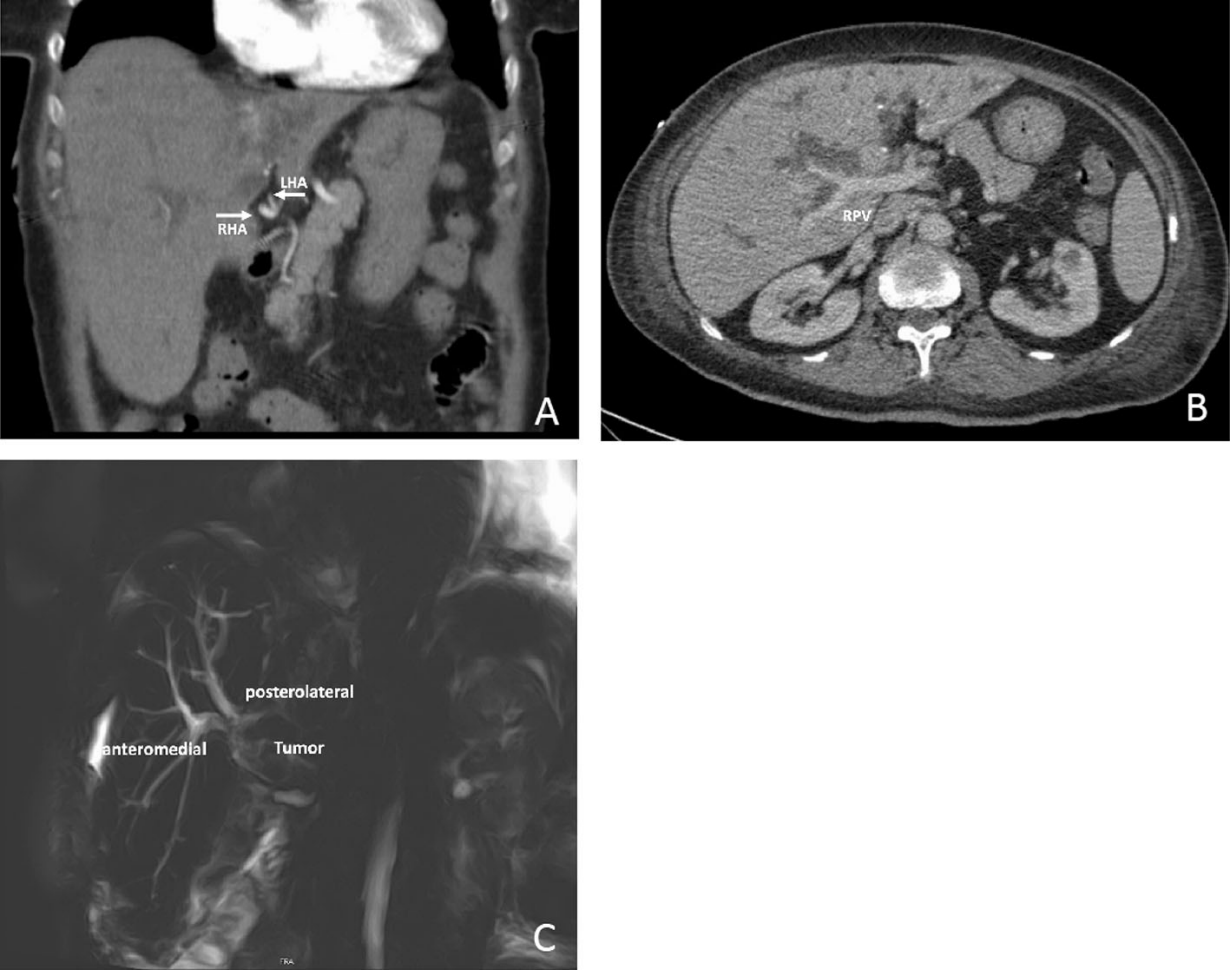
Preoperative computed tomography (CT) scan indicating no tumor infiltration of the **a** right hepatic artery (RHA) or **b** right portal vein (RPV). Magnetic resonance cholangiopancreatography (MRCP) suggests tumor-free anteromedial and posterolateral hepatic ducts. *LHA* left hepatic artery

As an example, we describe the case of a 78-year old woman with a pCCa type 3b who was scheduled for primary resection after recommendation of the interdisciplinary tumor board. Preoperative computer tomography (CT) suggested no tumor infiltration of the right hepatic artery or right portal vein including the bifurcation. Magnetic resonance cholangiopancreaticography (MRCP) suggested tumor-free right anteromedial and posterolateral hepatic ducts (Fig. 1). Liver function was assessed by the LiMAx test,^10^ which showed normal liver function (354 µg/kg/h), sufficient for a left hemihepatectomy. The patient received biliary drainage of the future right liver remnant before surgery according to the institutional guidelines.

## Results

### Surgical Procedure and Short-Term Outcome

**Fig. 2:**
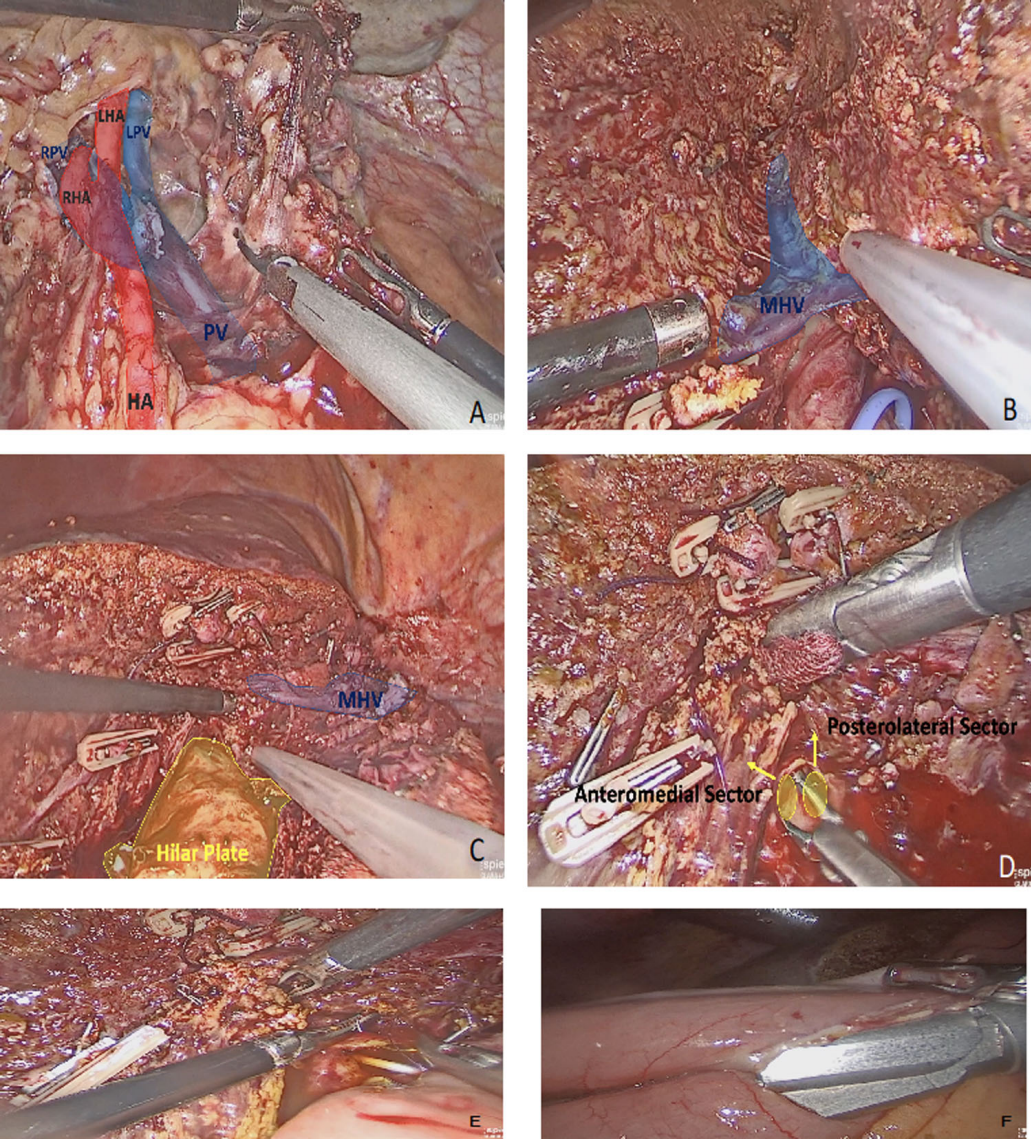
The main surgical steps for perihilar cholangiocarcinoma (pCCA) type 3b resection. **a** After dissection of the hepatic hilus, the portal vein (PV), left main branch of the portal vein (LPV), common hepatic artery (HA), right hepatic artery (RHA), and left hepatic artery (LHA) can be identified. **b** The middle hepatic vein (MHV) can be used as a landmark structure for anatomic resection. **c** Laparoscopic Caviton Ultrasonic Surgical Aspirator (CUSA) and ultrasonic shears are used for delicate preparation close to the hilar plate. **d** Tumor-free resection margins including intrahepatic biliary ducts are key to long-term survival for patients with pCCA. **e** Hepaticojejunostomy is performed after the insertion of two biliary drainages into the anteromedial and posterolateral sectors to splint the anastomosis, which is performed by running parachute sutures. **f** Foot-point anastomosis is performed with a stapler jejunojejunostomy

A detailed description of the surgical procedure is provided in the supplemental video, and Fig. 2 displays the major steps of the technique we use to perform left hepatectomy for patients with pCCA. In short, under general anesthesia and sterile conditions, the patient had surgery in a supine position with legs split and the surgeon standing between the legs. Hilar dissection allowed access to the hepatic hilum. This included identification of the common hepatic artery (HA), the left hepatic artery (LHA), the right hepatic artery (RHA), the portal vein (PV), and the left branch of the portal vein (LPV). Intraoperative frozen sections of the hilar lymph nodes at stations 8 and 12 showed no tumor infiltration (N0).

Removal of retroduodenal lymph nodes (station 13) was not performed in this case. After left vascular inflow control and ligature together with transection of the LHA and LPV, parenchymal transection was performed with preservation of the middle hepatic vein (MHV) as a landmark structure. As a standard procedure, the hepatic hilum is always loaded on a tape for an extracorporal Pringle maneuver. However, intermittent total vascular inflow occlusion is performed only if needed.

The laparoscopic Caviton Ultrasonic Surgical Aspirator (CUSA) and ultrasonic shears were used for parenchymal transection and delicate preparation close to the hilar plate. Vascular and biliary structures in the resection plane were clipped or ligated before transection. Wet bipolar coagulation was used to stop small bleedings. Intraoperative Doppler ultrasound was repeatedly applied for visualization of the vasculature and the tumor.

After transection of the right hepatic duct, frozen sections from the hepatic ducts of the anteromedial and posterolateral sector were obtained, showing tumor-free parenchymal resection margins (Fig. 2). The same was true for the distal resection margin of the resected common bile duct. Transection of bile duct structures is commonly performed after vascular transection to prevent early bile drainage into the operation site. To avoid local tumor seeding, biliary structures from the resected specimen are commonly ligated by running sutures after transection.

After mobilization of the caudate lobe, which for anatomic reasons must always be removed in a type 3b pCCA resection, the left hepatic vein was transected using a laparoscopic vascular stapler. The resected specimen was placed in a plastic bag and kept in the left upper abdomen until completion of biliary reconstruction. Biliary reconstruction was achieved with a Roux-en-Y retrocolic elevated jejunal segment.

Hepaticojejunostomy was performed as a total laparoscopic procedure with PDS 5-0 running sutures using a parachute technique to increase visibility during the suturing process. Biliary splints were inserted into both the anteromedial and posterolateral sectors before completion of the anastomosis. Gastrointestinal continuity finally was achieved with a distal stapler-jejunojejunostomy. The specimen was removed through an extended incision of the umbilical trocar access.

**Fig. 3:**
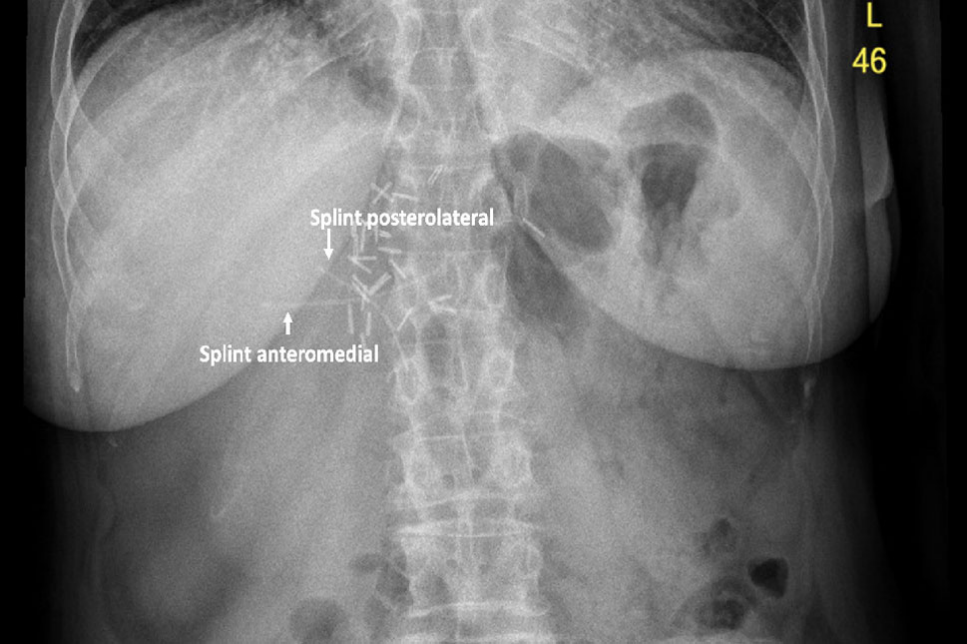
Internally draining biliary splints are placed by abdomen X-ray for visualization 3 weeks after surgery. Internal drainages, placed during hepaticojejunostomy, are released and excreted per via naturalis

The operation was completed in 386 min, with a total blood loss of less than 400 ml and no intra- or postoperative transfusion requirements. The patient received prolonged antibiotic treatment for clinically diagnosed cholangitis graded as a Clavien Dindo grade 2 complication. The patient was discharged on postoperative day 15. A 3-week follow-up x-ray of the abdomen displayed both biliary splints in situ (Fig. 3). The patient was scheduled for adjuvant chemotherapy after the final histopathologic grading of a G 2–3 adenocarcinoma, pT3 pN0 (0/8), M0, L1, V1, pN1, UICC IIIc R0.

## Discussion

Our surgical technique described in this report is a novel strategy for a hepaticojejunostomy performed totally by a minimally invasive procedure for patients with pCCA type 3b requiring major liver resection. Careful patient selection is key to successful surgical treatment, and from our perspective, laparoscopic extended left hepatectomy for pCCA type 3b especially can be feasible and safe if performed by specialized personnel. For reconstruction of the biliary drainage, performed totally by a minimally invasive procedure, the surgeon should aim for the lowest possible number of orifices. In open surgeries, we prefer an external biliary drainage, but for technical reasons, we place short perforated drains only for the splinting of the choledochojejunal anastomosis during a minimally invasive approach. The White test, a standardized procedure for intraoperative bile leakage testing,^11^ is not included in our minimally invasive protocol.

**Fig. 4:**
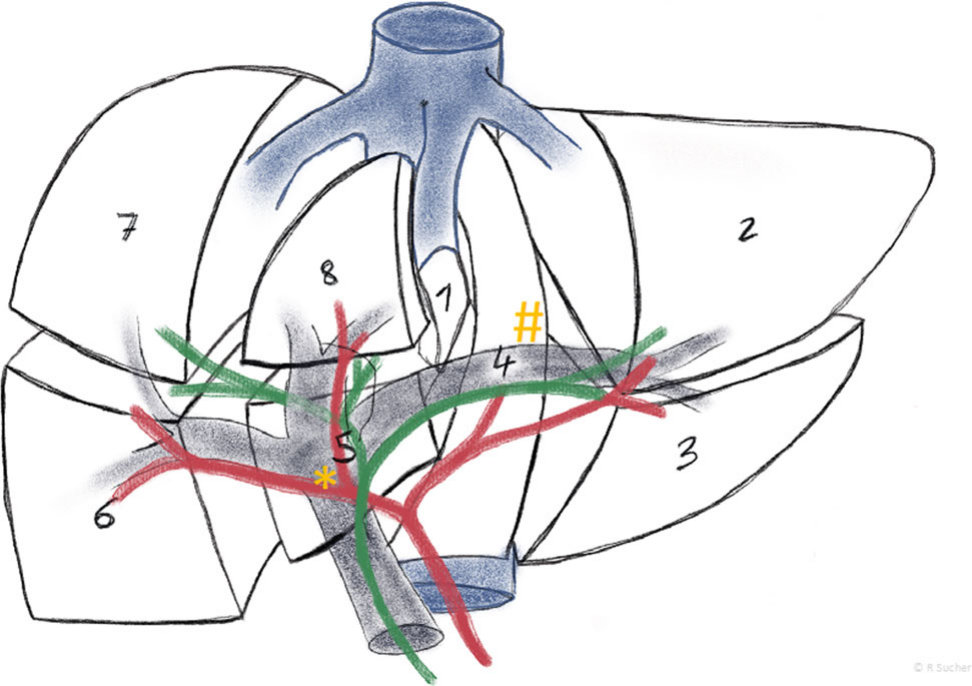
Anatomy of the liver according to Couinaud. A right trisectionectomy includes the removal of liver segments 1, 4, 5, 6, 7, and 8. Transection of the left hepatic duct (#), which varies in length from 1 to 5 cm, can commonly be performed before its segmentation into corresponding liver segments 2 and 3. In extended left hemihepatectomies or left trisectionectomies, which include the removal of liver segments [1, 2, 3, 4, 5, 8], the transection of right biliary structures (*) commonly requires reconstruction of several small orifices

From an anatomic perspective (Fig. 4), a more radical resection might be achieved on the right hilum than on the left hilum because the left hepatic duct, which varies in length from 1 to 5 cm, provides a more distant ramification and hence the opportunity for a more distant transection. However, “hilar on-block resection” which may provide oncologic superiority, requires both a right trisectionectomy and a portal vein resection.^12^ Laparoscopic portal vein resection and reconstruction might currently be very complex due to technical limitations, and the first data on robotic resections for pCCAs, with application of enhanced three-dimensional vison and unrestricted intracorporal maneuverability for delicate micro suturing, do not support its continued practice until significant technical and instrumental refinements are available.^13^ Vascular infiltration of pCCAs in the hepatic hilum currently can almost create a situation that hinders a laparoscopic oncologic resection. Enhanced recovery after surgery is key for pCCA patients to experience fast recovery and eventually receive timely adjuvant chemotherapy. However, further studies are required in the evolution of minimally invasive pCCA resections to move this procedure from an exploratory phase to a universally accepted and applied standard.

## Electronic supplementary material

Below is the link to the electronic supplementary material.

Supplementary material 1 (MOV 172873 kb)

## References

[1] Neuhaus P, Jonas S, Bechstein WO, Lohmann R, Radke C, Kling N, et al. Extended resections for hilar cholangiocarcinoma. *Ann Surg.* 1999;230:808–18; discussion 19. 10.1097/00000658-199912000-00010 PMC1420945 10615936

[2] Zhang Y, Dou C, Wu W, Liu J, Jin L, Hu Z (2020). Total laparoscopic versus open radical resection for hilar cholangiocarcinoma. Surg Endosc.

[3] Franken LC, van der Poel MJ, Latenstein AEJ, Zwart MJ, Roos E, Busch OR (2019). Minimally invasive surgery for perihilar cholangiocarcinoma: A systematic review. J Robot Surg..

[4] Ratti F, Fiorentini G, Cipriani F, Catena M, Paganelli M, Aldrighetti L (2020). Technical insights on laparoscopic left and right hepatectomy for perihilar cholangiocarcinoma. Ann Surg Oncol.

[5] Ratti F, Fiorentini G, Cipriani F, Catena M, Paganelli M, Aldrighetti L (2020). Perihilar cholangiocarcinoma: Are we ready to step towards minimally invasiveness?. Updates Surg..

[6] Cipriani F, Ratti F, Cardella A, Catena M, Paganelli M, Aldrighetti L (2019). Laparoscopic versus open major hepatectomy: analysis of clinical outcomes and cost effectiveness in a high-volume center. J Gastrointest Surg..

[7] Halls MC, Cipriani F, Berardi G, Barkhatov L, Lainas P, Alzoubi M (2018). Conversion for unfavorable intraoperative events results in significantly worse outcomes during laparoscopic liver resection: lessons learned from a multicenter review of 2861 cases. Ann Surg..

[8] Giuliante F, Ardito F, Guglielmi A, Aldrighetti L, Ferrero A, Calise F (2016). Association of lymph node status with survival in patients after liver resection for hilar cholangiocarcinoma in an Italian multicenter analysis. JAMA Surg..

[9] Ratti F, Fiorentini G, Cipriani F, Paganelli M, Catena M, Aldrighetti L (2019). Perioperative and long-term outcomes of laparoscopic versus open lymphadenectomy for biliary tumors: a propensity-score-based, case-matched analysis. Ann Surg Oncol..

[10] Stockmann M, Lock JF, Riecke B, Heyne K, Martus P, Fricke M (2009). Prediction of postoperative outcome after hepatectomy with a new bedside test for maximal liver function capacity. Ann Surg..

[11] Linke R, Ulrich F, Bechstein WO, Schnitzbauer AA (2015). The White test helps to reduce biliary leakage in liver resection: a systematic review and meta-analysis. Ann Hepatol..

[12] Neuhaus P, Thelen A, Jonas S, Puhl G, Denecke T, Veltzke-Schlieker W (2012). Oncological superiority of hilar en bloc resection for the treatment of hilar cholangiocarcinoma. Ann Surg Oncol..

[13] Xu Y, Wang H, Ji W, Tang M, Li H, Leng J (2016). Robotic radical resection for hilar cholangiocarcinoma: perioperative and long-term outcomes of an initial series. Surg Endosc..

